# Tetracycline resistance phenotypes and genotypes of coagulase-negative staphylococcal isolates from bubaline mastitis in Egypt

**DOI:** 10.14202/vetworld.2017.702-710

**Published:** 2017-06-25

**Authors:** K. A. Abd El-Razik, A. A. Arafa, R. H. Hedia, E. S. Ibrahim

**Affiliations:** 1 Department of Animal Reproduction, Veterinary Division, National Research Center, Dokki, Giza, Egypt; 2 Department of Microbiology and Immunology, Veterinary Division, National Research Center, Dokki, Giza, Egypt

**Keywords:** buffaloes, coagulase-negative staphylococci, mastitis, tetracycline resistance, *tet*K gene

## Abstract

**Aim::**

This study was devoted to elucidate the tetracycline resistance of coagulase-negative staphylococci (CNS) derived from normal and subclinical mastitic (SCM) buffaloes’ milk in Egypt.

**Materials and Methods: ::**

A total of 81 milk samples from 46 normal buffalo milk samples and 35 SCM buffalo milk samples at private dairy farms of Egypt were used in this study. CNS were identified using phenotypic and molecular methods (polymerase chain reaction [PCR]). CNS isolates were tested for tetracycline resistance using routine methods and multiplex PCR targeting tetracycline (*tet*) resistance genes followed by sequencing of positive PCR products and phylogenetic analysis.

**Results::**

Isolation and identification of 28 (34.5%) CNS from normal and SCM buffaloes’ milk, namely, *Staphylococcus intermedius* (39.2%), *Staphylococcus xylosus* (25.0%), *Staphylococcus epidermidis* (10.7%), *Staphylococcus hominis* (10.7%), and 3.5% to each of *Staphylococcus sciuri*, *Staphylococcus hyicus*, *Staphylococcus lugdunensis*, and *Staphylococcus simulans*. Using nested PCR, all the 28 CNS isolates revealed positive for 16srRNA gene specific for genus staphylococci and negative for thermonuclease (*nuc*) gene specific for *Staphylococcus aureus* species. The presence of tetracycline resistance-encoding genes (*tet*K, *tet*L, *tet*M, and *tet*O) was detected by multiplex PCR. All isolates were negative for *tet*L, M, and O genes while 14 (50%) CNS isolates were positive for *tet*K gene, namely, *S. lugdunensis* (100%), *S. hominis* (100%), *S. epidermidis* (66.6%), *S. intermedius* (45.4%), and *S. xylosus* (42.8%). Nucleotide sequencing of *tet*K gene followed by phylogenetic analysis showed the high homology between our CNS isolates genes of tetracycline resistance with *S. aureus* isolates including Egyptian ones. This proves the transfer of the tetracycline resistance encoding genes between coagulase-negative and coagulase positive *Staphylococcus* spp.

**Conclusion::**

CNS isolates have distinguishingly high resistance to tetracycline. Abundant tetracycline usage for mastitis treatment leads to the spread of genetic resistance mechanisms inside CNS strains and among all *Staphylococcus* spp. Consequently, tetracycline is not effective anymore.

## Introduction

Staphylococci are Gram-positive cocci-shaped bacteria that divided into coagulase-positive staphylococci and coagulase-negative staphylococci (CNS) based on the ability to coagulate rabbit plasma. CNS is characterized as non-*Staphylococcus aureus* staphylococci and considered as opportunistic mastitis pathogens. CNS has been generally viewed as minor pathogens. However, their significance has expanded in light of the fact that they have turned into the most frequently isolated group of species from bovine milk in numerous regions around the world and are regarded as emerging mastitis pathogens [1,2]. CNS usually causes subclinical mastitis (SCM), affects the quality of milk thus causing economic losses [3].

Antibiotic-resistant bacterial strains developed because of the boundless utilization of antibiotics on dairy farms and other food-producing animals [4] leading to severe public health issue that may be transmitted to human. Tetracycline-resistance genes (*tet*) appointed to classes K, L, M, and O have been recognized in staphylococci of animal origin [5]. Three mechanisms were confirmed to play a part in resistance to tetracycline in staphylococci [6].

The resistance genes may exchange from staphylococci of animal origin to staphylococci that cause infections in humans, in this way compromising antimicrobial treatment [7]. CNS that colonizing the udder of buffaloes and cows may represent a reservoir of different antibiotic-resistant genes. Screening the antimicrobial resistance of bacteria in the dairy industry should be performed [8].

Therefore, the aim of this study is to determine the prevalence and tetracycline resistance profiles of CNS species isolated from clinically normal and SCM in buffaloes, molecular identification of CNS, detection of tetracycline resistance genes using multiplex polymerase chain reaction (PCR), DNA sequencing, and phylogenetic analysis.

## Materials and Methods

### Ethical approval

The experiment was approved by Institutional Animal Ethics Committee.

### Sampling

A total of 81 milk samples were aseptically collected from 46 normal buffalo milk samples and 35 SCM buffalo milk samples at private dairy farms surrounding Giza and El-Beheira governorates of Egypt. The animals were not treated with any antibiotic for at least 30 days before samples collection. Ten to fifteen ml of milk from each quarter was manually collected into separate sterile 25 ml tubes. After gently suspending each sample, a collective milk sample representing all four quarters was created and quickly transported to the laboratory under chilled conditions and stored at 4°C until bacteriological and molecular examination.

### Isolation and identification of CNS [9,10]

One ml out of a 10 ml of each milk sample was mixed with 9 ml of tryptic soy broth and then, incubated for 8-12 h at 37°C. Ten microliters of milk were inoculated on tryptone soy agar plates supplemented with 5% bovine blood, which were incubated at 37°C for 18-24 h. All isolates were identified as CNS based on colony morphology, Gram-staining, catalase reaction, and oxidative–fermentative testing. After confirmation of the genus *Staphylococcus*, the enzyme coagulase was characterized among all isolates. Coagulase-negative isolates were subjected to identification to the species level using the API Staph commercial identification system (API Staph ID32 test; bioMérieux, Marcy l’Etoile, France).

### Antibiotic resistance assay

Tetracycline was selected for testing based on the licensing for mastitis treatment in cattle, use in human medicine and potential resistant determinant phenotypes [11,12]. Susceptibility of the isolates was determined against Tetracycline (30 μg) and the confirmed CNS isolates were inoculated into Mueller–Hinton broth (Oxoid) and incubated overnight at 37°C. The turbidity of the suspensions was adjusted to a 0.5 McFarland standard and streaked onto Mueller–Hinton agar (Oxoid) plates. Antimicrobial disks were added on the plates and they were incubated aerobically at 35°C for 16-18 h. The results were recorded as susceptible, intermediate, or resistant by measurement of the inhibition zone diameter according to the zone diameter interpretative standards of CLSI [13].

### Molecular confirmation of CNS identity

A crude DNA preparation was made from the 28 CNS isolates from milk samples. For extraction of DNA, bacterial pellets were re-suspended with 200 µl phosphate-buffered saline. DNA was extracted from isolates using the DNA extraction Kits (GF-1, Vivantis Co., Malaysia) according to manufacturer’s instructions. Duplex PCR was performed using two primer pairs [14,15], one pair targeting the *Staphylococcus* genus-specific 16S rRNA gene (fragment of 228 bp) and the second primer pair targeting the *S. aureus* specific *nuc* gene (fragment of 279 bp). In each executed PCR run, a positive *S. aureus* control (*S. aureus* DSM 20231 T), a positive CNS control (*Staphylococcus* *epidermidis* DSM 20044), and a negative control (water) were included for comparative analysis.

### PCR Screening of the genetic determinants of tetracycline resistance [16,17]

For PCR amplification reactions, a final volume of 50 μl contained 5 μl DNA templates; 25 μl of 2X Taq Master Mix (Cat. No. PLMM01, Vivantis Co., Malaysia). The primer concentrations were optimized for each multiplexed primer as follows: *tet*(K) 1.25 μΜ, *tet*(L) 1.0 μΜ, *tet*(M) 0.5 μΜ, and *tet*(O) 1.25 μΜ. An initial denaturation hot start of 5 min at 94°C was followed by 35 cycles consisting of 30 s of denaturation at 94°C, 30 s of annealing at 51°C and 30 s of extension at 72°C. Followed by a final extension of 10 min at 72°C. Isolates putatively containing genes encoding for tetracycline resistance were identified by comparison with positive controls. Amplicons were visualized after electrophoresis on a 2% agarose gel containing red safe (Table-1) [16,17].

**Table-1:** Primers used for detection of tetracycline sensitivity.

Gene	Primers (5’ > 3’)	Anneal. temp	Product	References
*tet*(K)	F; GTAGCGACAATAGGTAATAGT R; GTAGTGACAATAAACCTCCTA	51°C	360 bp	[16]
*tet*(L)	F; TCG TTA GCG TGC TGT CAT TC R; GTA TCC CAC CAA TGT AGC CG		267 bp	[17]
*tet*(M)	F; GTG GAC AAA GGT ACA ACG AG R; CGG TAA AGT TCG TCA CAC AC		406 bp	
*tet*(O)	F; AAC TTA GGC ATT CTG GCT CAC R; TCC CAC TGT TCC ATA TCG TCA		515 bp	

### DNA sequencing

PCR products were sequenced in MACROGEN Company (Korea) on 3730XL sequencers (Applied Biosystem, USA). The accuracy of data was confirmed by two-directional sequencing with the forward and reverse primers used in PCR.

The nucleotide sequences obtained in this study were analyzed using the BioEdit 7.0.4.1 and Muscle (http://www.ebi.ac.uk/Tools/msa/muscle/) programs. The resulting sequences were aligned with *tet*K gene of reference sequences of *Staphylococcus* spp. using a neighbor-joining analysis of the aligned sequences implemented in the program CLC Sequence Viewer 6. The reliability of the trees was estimated by bootstrap confidence values [18], and 500 bootstrap replications were used.

**Figure-1:**
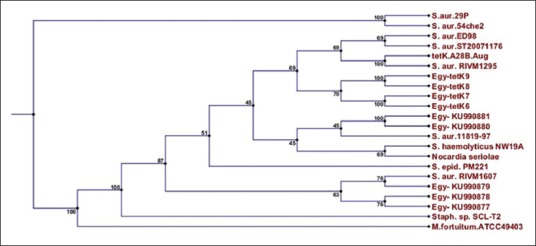
Phylogenetic relationship of selected strains of *Staphylococcus* spp. from different sources, representing the four distinct lineages, based on the *tet*K gene. The GenBank accession numbers of the isolates used are given.

The *tet*(k) of our isolates with sequences of 18 similar reference genes was used to construct the neighbor-joining tree (Figure-1) (by NCBI GenBank accession numbers) as shown in Table-2.

**Table-2:** Details of CNS strains used in the present study with the available ones in GenBank.

S.No.	Name	Strain	Source of isolation	Country	Access number
1	Egy-*tet*K6	*Staphylococcus lugudensis*	Buffaloes milk	Egypt	KX098498
2	Egy-*tet*K7	*Staphylococcus epidermidis*	Buffaloes milk	Egypt	KX098499
3	Egy-*tet*K8	*Staphylococcus xylosus*	Buffaloes milk	Egypt	KX098500
4	Egy-*tet*K9	*Staphylococcus hominis*	Buffaloes milk	Egypt	KX098501
5	Egy-KU990877	*Staphylococcus aureus*	Burger	Egypt	KU990877
6	Egy-KU990878	*Staphylococcus aureus*	Minced Beef	Egypt	KU990878
7	Egy-KU990879	*Staphylococcus aureus*	Bovine milk	Egypt	KU990879
8	Egy-KU990880	*Staphylococcus aureus*	Minced Beef	Egypt	KU990880
9	Egy-KU990881	*Staphylococcus aureus*	Bovine milk	Egypt	KU990881
10	*Staphylococcus haemolyticus* NW19A	*Staphylococcus haemolyticus* NW19A	Bovine milk	China	KM369884
11	*Staphylococcus epidermidis* PM221	*Staphylococcus epidermidis* PM221		Finland	NZ_HG813246
12	*Staphylococcus aureus* RIVM1607	*Staphylococcus aureus* RIVM1607	Human	Netherlands	CP013619
13	*Staphylococcus aureus* 29P	*Staphylococcus aureus* 29P	Pork samples collected from retail meat shops	India	KP658722
14	*Staphylococcus aureus* 54 Che2	*Staphylococcus aureus* 54 Che2	Chevon meat sample from retail shop	India	KP886833
15	*Staphylococcus aureus* ED98	*Staphylococcus aureus* ED98	Human	UK	CP001784
16	*Staphylococcus aureus* ST20071176	*Staphylococcus aureus* ST20071176	Human	Tunisia	KM281802
17	*Staphylococcus aureus* RIVM1295	*Staphylococcus aureus* RIVM1295	Human	Netherlands	CP013616
18	*tet*K_A28B_Aug	Uncultured bacterium	River benthic biofilm	New Zealand	KM668512
19	*Staphylococcus aureus* 11819-97	*Staphylococcus aureus* 819-97	Human (abscess on lower extremity)	Denmark	CP003193
20	*Staphylococcus* sp. SCL-T2	*Staphylococcus* spp.		New Zealand	JN801166
21	*Nocardia seriolae* CN101	*Nocardia seriolae* CN101	*Seriola dumerili* fish	Japan	AB513133
22	*Mycobacterium fortuitum* ATCC49403	*Mycobacterium fortuitum* ATCC49403		Korea	AF057465

CNS=Coagulase-negative staphylococci

### Nucleotide sequence accession numbers

Four sequences PCR samples (Egy-*tet*K 6-9) used in this study have been deposited in the GenBank database under accession no: KX098498, KX098499, KX098500, and KX098501, respectively.

## Results

### Bacterial isolation and identification

Our results confirmed the isolation and identification of 28 (34.5%) coagulase-negative staphylococci (CNS) from normal and SCM buffaloes’ milk (Table-3). Most of the isolates were *Staphylococcus intermedius* (39.2%) and *Staphylococcus xylosus* (25%), followed by *Staphylococcus hominis* and *S. epidermidis* (10.7% each), while the lowest incidences were for *Staphylococcus lugdunensis*, *Staphylococcus hyicus, Staphylococcus simulans* and *Staphylococcus sciuri* (3.5% each) as shown in Table-3.

**Table-3:** Prevalence of CNS species recovered from buffalo’s milk samples.

Health condition of the animals	No	*S. hyicus*	*S. epidermidis*	*S. hominis*	*S. xylosus*	*S. sciuri*	*S. lugdunensis*	*S. simulans*	*S. intermedius*	Total (%)
Healthy animal (normal milk samples)	46	1	2	2	3	0	1	1	6	16 (34.7)
Apparent normal animal (subclinical mastitic milk samples)	35	0	1	1	4	1	0	0	5	12 (34.2)
Total (%)	81	1 (3.5)	3 (10.7)	3 (10.7)	7 (25)	1 (3.5)	1 (3.5)	1 (3.5%)	11 (39.2)	28 (34.5)

CNS=Coagulase-negative staphylococci, *S. hyicus=Staphylococcus hyicus, S. epidermidis=Staphylococcus epidermidis, S. hominis=Staphylococcus hominis, S. xylosus=Staphylococcus xylosus, S. sciuri=Staphylococcus sciuri, S. lugdunensis=Staphylococcus lugdunensis, S. simulans=Staphylococcus simulans, S. intermedius=Staphylococcus intermedius*

### Phenotypic distribution of tetracycline resistance in CNS isolates

The *in vitro* sensitivity of the 28 CNS isolates for tetracycline revealed an incidence of resistance of 42.8% (12/28) in the CNS isolates as shown in Table-4, where 100% of *S. lugdunensis* and *S. hominis* and 66.6%, 42.8%, and 27.2% of *S. epidermidis*, *S. xylosus*, and *S. intermedius* isolates, respectively, were resistant to tetracycline. On the contrary, all the *S. hyicus*, *S. simulans*, and *S. sciuri* isolates were sensitive to tetracycline antibiotic.

**Table-4:** Distribution of tetracycline resistance gene combinations in the 7 different CNS species constituting the 28 CNS isolates from buffalo milk samples.

*Staphylococcus* species identified by API	No of isolates	Tetracycline resistance assay	Antibiotic resistance gene (*tet*K)
*S. intermedius*	3	R	+
*S. intermedius*	5	S	-
*S. intermedius*	1	S	+
*S. intermedius*	1	I	+
*S. intermedius*	1	I	
*S. xylosus*	3	R	+
*S. xylosus*	4	S	-
*S. epidermidis*	2	R	+
*S. epidermidis*	1	S	-
*S. hominis*	3	R	+
*S. lugdunensis*	1	R	+
*S. hyicus*	1	S	-
*S. simulans*	1	S	-
*S. sciuri*	1	S	-

R=Resistant, I=Intermediate, S: Susceptible, CNS=Coagulase-negative staphylococci, *S. hyicus=Staphylococcus hyicus, S. epidermidis=Staphylococcus epidermidis, S. hominis=Staphylococcus hominis, S. xylosus=Staphylococcus xylosus, S. sciuri=Staphylococcus sciuri, S. lugdunensis=Staphylococcus lugdunensis, S. simulans=Staphylococcus simulans, S. intermedius=Staphylococcus intermedius*

### Nested PCR targeting 16S rRNA gene and nuc gene of 28 CNS isolates

The results revealed a positive amplification of 228 bp fragment of primer specific for 16s rRNA gene (specific for genus *Staphylococcus*) and a negative amplification of *nuc* gene at 279 bp (*S. aureus* species specific) for all 28 isolates of the genus staphylococci examined.

### Results of multiplex PCR for the genes encoding tetracycline

**Figure-2:**
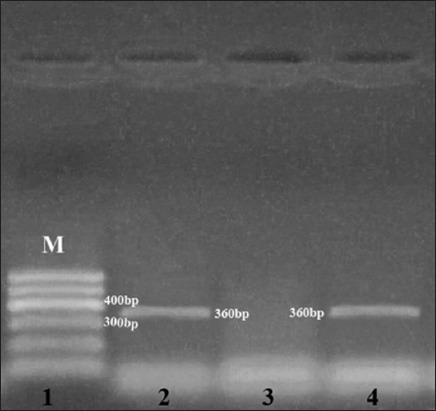
Agarose gel electrophoresis using polymerase chain reaction with amplification of the 360-bp fragment for the tetracycline resistance (*tet*K) gene performed with specific primer: Lane 1, 100-bp DNA ladder; lane 2, positive control: Lane 3, negative control, lane 4, positive amplification of the 360 bp fragment of the *tet*K gene from coagulase-negative staphylococci species.

Using multiplex PCR, all isolates were negative for *tet*L, M, and O genes encoding tetracycline while *tet*K gene was detected in 14 CNS isolates (Figure-2) with an incidence of 50%. In detail, the *tet*K gene was detected in all *S. lugdunensis* and *S. hominis* isolates with an incidence of 100% while it was detected in *S. epidermidis*, *S. intermedius*, and *S. xylosus* with an incidence of 66.6%, 45.4%, 42.8%, respectively, as shown in Table-4. On the contrary, all the *S. hyicus*, *S. simulans*, and *S. sciuri* isolates were negative to for *tet*K gene.

### Association of antimicrobial resistance phenotype with resistance-associated genes

Analysis of the presence of the *tet*K genes in the 28 CNS isolates with antimicrobial resistance patterns was conducted as shown in Table-4. A detailed analysis displayed associations of resistance/susceptibility phenotypes with potential resistance genes except in two isolates where an intermediate resistance phenotype (1/11, 9.09%) and a susceptible phenotype (1/11, 9.09%) harbored the *tet*K gene.

### Phylogenetic analysis

**Figure-3:**
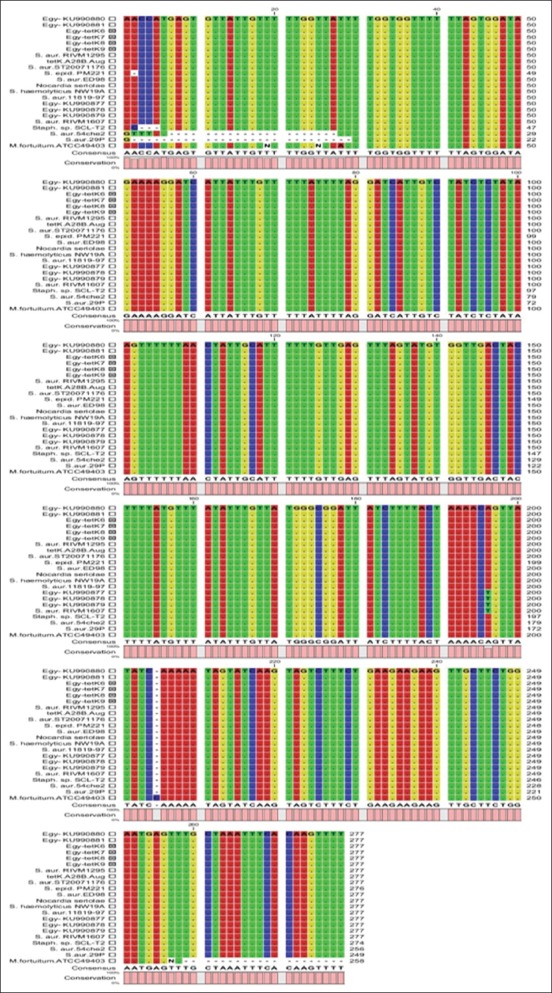
Multiple sequence alignment of coagulase-negative staphylococci (present study) isolated from buffaloes milk (Egy-*tet*K 6-9) and their corresponding reference sequences. Only variable sites are shown with different color. Dashes in the middle indicate gaps.

The phylogenetic tree showed that all *tet*(K) genes of tetracycline resistance (Egy-*tet*K 6-9) were gathered with their similar reference genes sequences and formed two groups, which indicating high level of identity between the local isolates genes and their corresponding reference sequences in the GenBank (Figure-3).

### Sequence analysis of *tet*(K) gene

The sequences of *tet*(K) genes of CNS obtained in the current study were compared with the sequences of *tet*(K) genes retrieved from GenBank. Similarity between obtained sequences with those from GenBank was 69.00 to 100%. This showed that the sequenced part of *tet*(K) gene was from a highly conservative region (Figure-3). The only change was the (A) nucleotide number 196 was substituted with (T) base in the Egyptian *S. aureus* isolates (Ku-990877-79) and RIVM1607. Phylogenetic tree of the *tet*K gene sequences was inferred using the maximum likelihood method based on Tamura-Nei model (24). Based on generated phylogenetic tree, four CNS isolates examined in the present study grouped in seven distinct clusters (Figure-1). Phylogenetic analysis confirmed the results of PCR for the four CNS isolates.

## Discussion

Coagulase-negative staphylococci have been considered the most common mastitis causing agents in several countries [19]. They mostly cause SCM (Pyörälä and Taponen, 2009). CNS mastitis responds much better to antimicrobial treatment than *S. aureus* mastitis but it is realized that resistance to various antimicrobials is more prevalent in CNS than in *S. aureus* [20] as they can easily develop multi-resistance.

Our results confirmed the isolation and identification of 28 (34.5%) CNS from normal and SCM buffaloes’ milk (Table-3). Most of the isolates were *S. intermedius* (39.2%) and *S. xylosus* (25%). This was in agreement with that of Osman *et al*. [21], with an incidence of *S. intermedius* (31.8%) and *S. xylosus* (29.5%), respectively.

Moreover, this was also comparable to that of El-Ashker *et al*. [22] that confirmed an isolation rate of 44.4%CNS with *S. xylosus* as the most prevalent CNS from buffalo’s milk (75%). Although *S. xylosus* is not known to cause mastitis, this, emphasizing previous studies that *S. xylosus* is an underestimated pathogenic CNS in bovine mastitis [3].

Not very many studies have examined contrasts in antimicrobial resistance among CNS species [10]. Distinguishing to species level would be critical in the event that it has effect on administration and treatment choices [23].

From the current study, the *in vitro* sensitivity of the 28 CNS isolates against tetracycline revealed an incidence of resistance of 42.8% in the CNS isolates, where 100% of *S. hominis* and *S. lugdunensis* and 66.6%, 42.8% and 27.2% of *S. epidermidis, S. xylosus* and *S. intermedius* isolates, respectively, were resistant to tetracycline. On the contrary, all the *S. hyicus, S. simulans* and *S. sciuri* isolates were sensitive to tetracycline antibiotic. This was in agreement to that of Osman *et al*. [8] which revealed an incidence of tetracycline resistance of 25.5% in the CNS isolates, where the 75% of *S. hominis*, 100% of *S. lugdunensis* and 50%, 23% and 21.4% of *S. epidermidis, S. xylosus* and *S. intermedius* isolates, respectively, were resistant to tetracycline. The other isolates were typically sensitive to tetracycline as in our results.

Our results can be explained that, in most countries, tetracycline is routinely used to treat mastitis and in the water of the herd as a prophylactic measure aimed at reducing infections [24]. Widespread and continuous use of tetracycline leads to increase in resistance toward these antimicrobial agents [25].

Using PCR, positive amplification of 16srRNA gene fragment (specific for genus *Staphylococcus*) and a negative amplification of *nuc* fragment for all isolates (*S. aureus* species specific). This proves that genotypic methods have higher specificity and sensitivity than other methods for discriminating among species, resulting in a better alternative for the routine identification of CNS isolates as reported by [26].

Tetracycline resistance determinants are broad among bacterial species and are regularly found in multi-drug resistant bacteria [27]. Resistance is frequently due to the obtaining of new genes connected with either conjugative plasmids or transposons [6]. Tetracycline is utilized for treatment of bovine mastitis [28]. Prolonged use may prompt to the emergence of tetracycline resistant *Staphylococcus* species which is a serious concern not only in animal health but also to human health because of the presence of tetracycline resistant genes which can be exchanged between staphylococcal species through horizontal exchange and these pathogens harboring resistant genes can be transferred to humans from bovines and vice versa [29,30].

In the current study, we identified the tetracycline resistant genes *tet*(K), (L), (M), and (O) associated with a ribosomal protection mechanism and/or efflux mechanism [6]. The recognition of tetracycline resistance genes may be utilized as an extra genotypic marker for outbreak investigation and surveillance as reported by Duran *et al*. [16] and Ng *et al*. [17]. The used multiplex PCR has appeared to be a helpful technique to differentiate the mechanisms of tetracycline resistance.

Using multiplex PCR, all isolates were negative for *tet*L, M, and O genes while *tet*K gene was detected in 14 (50%) CNS isolates. The *tet*K gene was detected in all *S. lugdunensis* and *S. hominis* isolates with an incidence of 100% while it was detected in *S. epidermidis*, *S. intermedius*, and *S. xylosus* with an incidence of 66.6%, 45.4%, and 42.8%, respectively. This was parallel to that of Osman *et al*. [8] with the detection of the *tet*K gene in 28 (29.8%) of CNS isolates (*S. lugdunensis* [100%], *S. hominis* [75%], *S. epidermidis* [40%], *S. xylosus*, [28.6%] and *S. intermedius* [26.7%]). In contrary to that El-Ashker *et al.*, [22] using PCR for CNS isolated from buffaloes milk in Egypt. CNS isolates were positive to *tet*(M) gene and negative for *tet*(K) gene.

Differences between Egypt and other countries in antimicrobial usage could be contributed to differences in resistance gene profiles of CNS isolates originating from the same host species in different countries [10,31,32]. On account of mastitis-causing CNS, it is critical to identify resistant strains because such strains can serve as a store of resistance genes that can be exchanged to other bacteria posing additional difficulties to the control and cure of ­mastitis [7] and that could potentially pose a human health hazard [23].

From our previous results, it is clear that the rising incidence of resistance- encoding genes is usually related to long-term usage of tetracycline to treat various infections in the veterinary field as confirmed by Klimiene *et al*. [33].

A detailed phenotypic (42.8%) and genotypic (50%) tetracycline resistance analysis displayed associations of resistance/susceptibility phenotypes with potential resistance genes except in two isolates where an intermediate resistance phenotype (1/11, 9.09%) harbored the *tet*K determinant and a susceptible phenotype (1/11, 9.09%) harbored the *tet*K determinant. This was in contrary to Cengiz *et al*. [34] who reported that phenotypically resistance tetracycline strains were more pervasive when contrasted with genotypically resistance strains in which only 33.3% out of the phenotypically resistance *S*. *aureus* strains demonstrates the presence of tetracycline (*tet*K/*tet*M) resistance gene.

The multiple sequence alignment was done using different bioinformatics softwares, the results obtained showed high level of similarities (homology) between the local isolates sequences of *tet*(k) and the reference sequences which retrieved from the GenBank databases including *S. aureus* strains, especially Egyptian (NCBI GenBank, http://www.ncbi.nlm.hih.gov/) except minor variations (Figure-3). This proves the possibility of transfer of the tetracycline resistance encoding genes between coagulase-negative and coagulase-positive *Staphylococcus* spp.

## Conclusion

CNS isolates have distinguishingly high resistance rates to tetracycline. Abundant tetracycline usage for mastitis treatment leads to the spread of genetic resistance mechanisms inside CNS strains and among all *Staphylococcus* spp. Consequently, tetracycline is not effective anymore due to the high resistance rates in CNS isolated from buffalo cows with SCM or even clinically normal. Further, studies are needed to investigate the presence of other genes responsible for tetracycline resistance and this can be done using large number of samples and sequenced through the whole genome sequence to get complete picture on these genes.

## Authors’ Contributions

KAA conceived and designed the work performed the phylogenetic analysis and wrote the manuscript. AAA conducted the research work regarding the antibiotic resistance, molecular techniques, and the analysis and assisted in writing of the manuscript. Both RHH and ESI have assisted this research work regarding sampling, bacterial isolation, and identification. All authors read and approved the final manuscript.
